# Microbiome-targeted Alzheimer’s interventions via gut-brain axis

**DOI:** 10.3389/fmicb.2025.1729708

**Published:** 2025-12-05

**Authors:** Ruiqi Qin, Chaofan Li, Xingxing Yuan, Yinghua Chen

**Affiliations:** 1 Department of Graduate School, Heilongjiang University of Chinese Medicine, Harbin, Heilongjiang, China; 2 The Fifth Department of Acupuncture, First Affiliated Hospital of Heilongjiang University of Chinese Medicine, Harbin, Heilongjiang, China; 3 Department of Medicine, Heilong Academy of traditional Chinese Medicine, Harbin, Heilongjiang, China

**Keywords:** gut-brain axis, Alzheimer’s disease, microbiome dysbiosis, neuroinflammation, short-chain fatty acids

## Abstract

Alzheimer’s disease (AD) is a progressive neurodegenerative disorder with limited treatment options, underscoring the need for novel therapeutic targets. The gut-brain axis has emerged as a critical bidirectional communication system, with growing evidence establishing gut dysbiosis as a causal factor in AD pathogenesis. This dysbiosis, characterized by a reduction in beneficial microbes and an increase in pro-inflammatory taxa, compromises intestinal and blood–brain barrier integrity, promoting systemic inflammation and the translocation of neurotoxic agents like lipopolysaccharide (LPS). Consequently, the balance of key microbial metabolites is disrupted, reducing neuroprotective short-chain fatty acids (SCFAs) and indoles while elevating inflammatory mediators, which collectively exacerbate neuroinflammation, amyloid-*β* (Aβ) deposition, and tau pathology. This review evaluates promising interventions, including probiotics, anti-inflammatory diets, exercise, and phytochemicals that can restore microbial balance, enhance barrier function, and improve cognitive outcomes in preclinical and early clinical studies. However, clinical translation is hindered by an overreliance on animal models, short-term studies, and insufficient mechanistic insight. Future research must prioritize large-scale human trials, multi-omics integration to elucidate signaling pathways, and personalized approaches that account for host genetics and baseline microbiome composition to fully harness the therapeutic potential of the gut-brain axis for AD.

## Graphical abstract

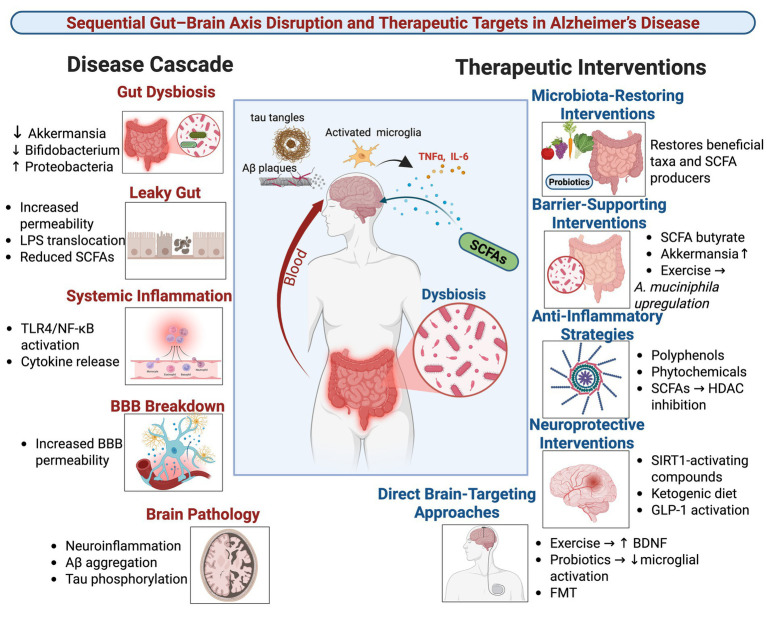

## 1 Introduction

Alzheimer’s disease (AD) is a progressive neurodegenerative disorder with limited effective treatment options, driving the search for novel pathogenic mechanisms and therapeutic targets. Emerging research underscores the gut-brain axis as a critical bidirectional communication system that plays a fundamental role in the pathogenesis and progression of AD and related cognitive decline (Sun M. et al., 2020; Denman et al., 2023; Su et al., 2025). This system integrates neural, endocrine, immune, and metabolic signals between the gut microbiota and the central nervous system (CNS) (McGrattan et al., 2019; Grabrucker et al., 2023). A characteristic gut dysbiosis, marked by an increase in Proteobacteria and Clostridia alongside a decline in beneficial taxa such as Akkermansia, Blautia, and *Clostridium butyricum*, is consistently observed in both AD patients and experimental models, correlating with cognitive deficits and AD-related neuropathology (Varesi et al., 2022; Marizzoni et al., 2023). While compelling, the current evidence base derives predominantly from animal studies, with human clinical data often limited by smaller sample sizes, shorter durations, and methodological heterogeneity. Furthermore, confounding factors inherent to human studies; such as diverse dietary patterns, concomitant medications, and comorbidities, complicate the establishment of direct causality and highlight the need for more rigorous, controlled human trials.

The mechanisms through which gut dysbiosis contributes to AD pathology are becoming increasingly clear, though a deeper mechanistic integration is required. Dysbiosis compromises intestinal barrier function, leading to a “leaky gut” that permits the translocation of microbial products like LPS into systemic circulation (Yang X. et al., 2020; Gates et al., 2022). This promotes systemic inflammation and increases blood–brain barrier (BBB) permeability, facilitating the entry of neurotoxic agents into the CNS. Concurrently, dysbiosis alters the production of key microbial metabolites. A reduction in neuroprotective short-chain fatty acids (SCFAs), particularly butyrate, coupled with elevated neurotoxic metabolites such as quinolinic acid and a decrease in anti-inflammatory mediators like IL-10, exacerbates neuroinflammation. These effects are mediated through the activation of microglia and engagement of signaling pathways involving NF-κB and the NLRP3 inflammasome (Erny et al., 2021; Amin et al., 2023; Nakhal et al., 2024). Critically, microbial metabolites also signal through specific host receptors, including free fatty acid receptors (FFAR2/3) for SCFAs, Toll-like receptor 4 (TLR4) for LPS, and the aryl hydrocarbon receptor (AhR) for tryptophan derivatives like indoles to modulate neuroimmune responses and neuronal integrity (Marizzoni et al., 2020; Chen S. et al., 2023). The causal role of gut microbiota in AD is further reinforced by studies demonstrating that fecal microbiota transplantation (FMT) from AD patients into healthy rodents can induce cognitive impairments and deficits in hippocampal neurogenesis (Bonfili et al., 2022; Fei et al., 2023).

Considering these mechanistic insights, therapeutic strategies targeting the gut-brain axis show considerable promise. Interventions such as anti-inflammatory dietary patterns (Mediterranean or DASH diets), specific probiotics (*Clostridium butyricum*), physical exercise, and phytochemicals have been shown in preclinical and early clinical studies to restore microbial balance, attenuate neuroinflammation, improve synaptic function, and enhance cognition (Hao et al., 2024; Nicolas et al., 2024; Zheng et al., 2024). However, the translation of these findings is complicated by the influence of host factors such as genetics, baseline microbiome composition, diet, medications, and comorbidities, which can significantly modulate individual responses to intervention.

This review aims to synthesize current evidence on the causal role of gut dysbiosis in AD, critically evaluate the promise and limitations of microbiome-targeted therapies, and assess the key knowledge gaps that must be addressed to advance gut-brain axis-based interventions for the prevention and treatment of AD. We place a particular emphasis on the need to bridge preclinical findings with human clinical evidence and to elucidate the complex, interlinked signaling pathways that mediate gut-brain communication in AD.

## 2 Biological mechanisms in targeting the gut-brain axis in AD

AD is a progressive neurodegenerative disorder characterized by the accumulation of Aβ plaques, neurofibrillary tangles, chronic neuroinflammation, synaptic dysfunction, and neuronal loss (Knopman et al., 2021; Kamatham et al., 2024). While existing pharmacological treatments show limited efficacy, non-pharmacological approaches targeting the gut-brain axis have emerged as promising therapeutic avenues. The foundational element of this axis is gut microbiota dysbiosis, which manifests as reduced microbial diversity, a decrease in beneficial bacteria such as Bifidobacterium and Akkermansia, and an increase in pro-inflammatory taxa like Proteobacteria (Loera-Valencia et al., 2019; Sharma et al., 2021; Dhanawat et al., 2025). This section first systematically outlines the biological cascade through which gut dysbiosis drives AD pathology and then evaluates how various interventions can counteract this cascade.

### 2.1 The disease cascade: from gut dysbiosis to neural insult

The pathogenic sequence begins with gut dysbiosis, which initiates a multi-step cascade culminating in the hallmark pathologies of AD.

#### 2.1.1 Barrier disruption and systemic inflammation

Dysbiosis disrupts intestinal barrier integrity, resulting in a “leaky gut” that permits the translocation of bacterial endotoxins, most notably LPS, into systemic circulation (Gates et al., 2022; Ticinesi et al., 2022). LPS and other inflammatory mediators promote a state of chronic systemic inflammation. This systemic inflammation, in turn, compromises the BBB, allowing neurotoxic agents to enter the CNS. Once in the brain, LPS activates microglia and astrocytes primarily through the TLR4/NF-κB signaling pathway, triggering a robust neuroinflammatory response (Li et al., 2024; Kamila et al., 2025).

#### 2.1.2 Metabolite imbalance and key signaling pathways

Concurrently, dysbiosis disrupts the production of key microbial metabolites, reducing neuroprotective compounds while increasing neurotoxic ones.

*SCFAs*: SCFAs (acetate, propionate, butyrate), produced by bacterial fermentation of dietary fiber, are often depleted in AD. They exert anti-inflammatory and neuroprotective effects by inhibiting histone deacetylases (HDACs) to enhance synaptic plasticity genes (BDNF, SYN1) and by serving as ligands for free fatty acid receptors (FFAR2/3). Activation of FFAR2/3 on intestinal cells stimulates the release of glucagon-like peptide-1 (GLP-1), which can reduce neuroinflammation and improve insulin sensitivity (Bonfili et al., 2017; Zhang B. et al., 2022; Gasmi et al., 2025).

*Tryptophan metabolites*: Gut microbes metabolize tryptophan into bioactive indole derivatives (indole-3-acetic acid) and acid. These metabolites can activate the aryl hydrocarbon receptor (AhR), a key regulator of immune responses and barrier integrity. A dysbiosis-induced reduction in these AhR agonists contributes to increased oxidative stress, impaired gut barrier function, and heightened neuroinflammation (Zhang Z. et al., 2022; Chen S. et al., 2023; Jin et al., 2023).

#### 2.1.3 Neuroinflammatory amplification and AD pathology

The influx of systemic inflammatory signals and the deficit of anti-inflammatory metabolites promote the polarization of microglia toward a pro-inflammatory “MGnD” phenotype. These activated microglia release cytokines such as TNF-*α*, IL-1β, and IL-6, which further enhance Aβ production via BACE1 upregulation and promote tau hyperphosphorylation through GSK-3β activation (Wei et al., 2023; Zhao et al., 2023; Li et al., 2024). This creates a vicious cycle of neuroinflammation that directly fuels the core pathological triad of AD: Aβ accumulation, tau pathology, and neuronal loss.

### 2.2 Therapeutic interventions targeting the gut-brain axis

The mechanistic cascade described above provides a clear roadmap for interventions aimed at restoring gut-brain axis homeostasis. The following strategies have shown promise in preclinical and early clinical studies.

#### 2.2.1 Probiotics and prebiotics

Specific probiotic strains directly modulate the gut-brain axis to ameliorate AD pathology. For example, *Lactobacillus* and *Bifidobacterium* strains have been shown to reduce Aβ plaques and tau phosphorylation by enhancing SCFA production and suppressing LPS-induced TLR4/NF-κB activation (Mosaferi et al., 2021b; Cox et al., 2022; Zhang S. et al., 2022). *Clostridium butyricum* is a notable butyrate-producing probiotic that ameliorates Aβ burden and microglial activation (Sun J. et al., 2020). Prebiotics, such as fructooligosaccharides and inulin, serve as fuel for these beneficial bacteria, promoting their growth and metabolic activity, thereby indirectly conferring neuroprotective effects.

#### 2.2.2 Polyphenols and phytochemicals

Dietary phytochemicals exert multi-targeted effects. Resveratrol activates SIRT1 to suppress NF-κB signaling, while scutellarin modulates cAMP-PKA-CREB-HDAC3 signaling in microglia (Grinan-Ferre et al., 2021; Zhang S. et al., 2022). Flavonoids like isoorientin and cyanidin-3-O-glucoside (C3G) reshape the gut microbiota composition, decreasing the abundance of pro-inflammatory taxa, reducing circulating LPS and TNF-α, and improving gut barrier function (Zhang Z. et al., 2022; Oumeddour et al., 2024). Many of these compounds also possess direct antioxidant properties.

#### 2.2.3 Dietary interventions

Comprehensive dietary patterns offer a powerful approach to modulating the gut ecosystem.

*Mediterranean and high-fiber diets*: These diets increase the abundance of SCFA-producing bacteria (*Bacteroides*), thereby elevating circulating SCFA levels, which is associated with lower Aβ and tau pathology (Gregory et al., 2023; Oumeddour et al., 2024).

*Ketogenic diets*: By shifting the body’s primary energy source to fats, ketogenic diets elevate neuroprotective ketone bodies and have been shown to enhance neurovascular function (Ma et al., 2018; Nanda et al., 2024).

*Time-restricted eating:* This intervention enriches beneficial mucin-degrading bacteria like *Akkermansia* and helps restore circadian rhythms, which are often disrupted in AD (Gasmi et al., 2025).

#### 2.2.4 Physical exercise

Regular physical activity is a potent non-pharmacological intervention that boosts levels of *Akkermansia muciniphila*, enhances BBB integrity, increases the expression of brain-derived neurotrophic factor (BDNF), and reduces gut permeability, thereby preventing the translocation of LPS into the circulation (Rosa et al., 2020; Mengoli et al., 2023).

#### 2.2.5 Fecal microbiota transplantation (FMT)

FMT from healthy donors represents the most comprehensive approach to resetting the gut microbiome. Studies demonstrate that FMT can restore microbial diversity and SCFA production, suppress pro-inflammatory microglia, promote Aβ clearance, and improve cognitive function in AD models, providing direct evidence for the causal role of the gut microbiota (Grabrucker et al., 2023; Jin et al., 2023).

### 2.3 The influence of host factors and comorbidities

The efficacy of the interventions described above is not uniform and is significantly influenced by host factors. Gut microbiota composition and its associated cognitive impacts can vary by sex, as demonstrated by studies where the probiotic VSL#3 reduced Aβ plaques only in female APP/PS1 mice (Kaur et al., 2021). Furthermore, common comorbidities such as obesity and type 2 diabetes mellitus (T2DM) exacerbate gut dysbiosis and systemic inflammation, thereby accelerating AD progression (Bonfili et al., 2020; Zhang S. et al., 2022). This underscores the necessity for personalized approaches that account for baseline gut microbiota composition, diet, sex, genetics, and co-existing medical conditions to optimize treatment response (Mosaferi et al., 2021a; Jiang et al., 2022; Chatterjee et al., 2024; Hediyal et al., 2024).

In summary, gut dysbiosis initiates a cascade involving barrier disruption, metabolite imbalance, and neuroinflammation that drives AD pathology. Non-pharmacological strategies counter this cascade through interconnected biological mechanisms that involve restoring gut microbial balance, enhancing gut and BBB integrity, increasing neuroprotective metabolites, and suppressing neuroinflammation via specific pathways such as FFAR2/3, TLR4, and AhR. Future research must prioritize human studies and personalized regimens to effectively halt AD progression Table 1.

**Table 1:** Summary of microbiome-targeted intervention studies in Alzheimer’s disease.

Study objective	Study design	Main findings	Conclusion	Reference
To investigate if microbiota from AD patients can induce cognitive deficits.	FMT from AD patients or healthy controls into antibiotic-treated adult rats. Behavioral and histological analysis.	FMT from AD donors impaired cognitive function and reduced adult hippocampal neurogenesis in recipient rats compared to FMT from healthy donors.	Gut microbiota dysbiosis in AD patients plays a causal role in cognitive deficits, transferable via FMT.	Grabrucker et al. (2023)
To assess the cognitive effects of a probiotic intervention in older adults with Mild Cognitive Impairment (MCI).	A randomized, controlled clinical trial where older adults with MCI received a multi-strain probiotic or placebo for 12 weeks.	The probiotic group showed significant improvements in multiple cognitive domains compared to the placebo group.	Probiotic intervention can benefit neural function and improve cognitive performance in older adults with MCI.	Fei et al. (2023)
To test a multi-strain probiotic formulation (SLAB51) in an AD mouse model.	APP/PS1 mice were administered SLAB51 orally. Analyzed brain pathology, inflammation, and gut microbiota composition.	SLAB51 restored microbial balance, increased SCFA levels, reduced brain Aβ aggregates, and decreased neuroinflammation.	Probiotic SLAB51 modulates the gut-brain axis, countering AD progression by influencing proteolysis and inflammation.	Markulin et al. (2022)
To evaluate the impact of forced treadmill running on gut microbiota and AD pathology.	3xTg-AD mice underwent forced treadmill running. Assessed cognition, Aβ/tau pathology, and gut microbiome.	Exercise alleviated cognitive impairment and AD pathology, which was associated with beneficial shifts in gut microbiota composition.	Exercise-induced modifications of gut microbiota contribute to the alleviation of cognitive decline and AD pathology.	Zhang B. et al. (2022)
To examine the effect of probiotic *Clostridium butyricum* on AD pathology.	APP/PS1 transgenic mice were treated with *C. butyricum* for 8 weeks. Assessed behavior, pathology, and gut microbiota.	Treatment improved cognitive function, reduced Aβ plaques and neuroinflammation, and increased beneficial gut bacteria and butyrate levels.	*C. butyricum* supplementation ameliorates AD pathology via the microbiota-gut-brain axis, likely through butyrate.	Nanda et al. (2024)
To determine the role of *Akkermansia muciniphila* in cognitive function and amyloid pathology.	APP/PS1 mice were treated with live *A. muciniphila* for 8 weeks. Cognitive tests and brain analysis were performed.	*A. muciniphila* treatment improved cognitive performance and reduced Aβ plaque load in the cortex and hippocampus.	Supplementation with *A. muciniphila* could be a novel probiotic strategy for preventing or delaying AD progression.	Westfall et al. (2019)
To explore the protective effects of a synbiotic in a *Drosophila* model of AD.	AD-model flies were fed a novel synbiotic. Lifespan, climbing ability, and Alzheimer’s-related markers were assessed.	The synbiotic delayed the onset of AD symptoms and extended lifespan via combinatorial gut-brain-axis signaling.	Synbiotics represent a promising non-pharmacological approach to delay AD onset through gut-brain signaling.	Kim et al. (2022)

Selected studies were chosen to represent a diversity of intervention types (probiotics, FMT, exercise, synbiotics), study models (rodents, flies, human trials), and key mechanistic insights (SCFA restoration, microglial modulation, gut barrier repair) that are central to the themes discussed in this review.

## 3 Study limitations and research gaps in targeting the gut-brain axis in AD

Non-pharmacological interventions targeting the gut-brain axis represent a promising strategy for modulating AD progression. However, their clinical translation is impeded by substantial limitations in current research and critical knowledge gaps. This section provides a critical evaluation of these issues, emphasizing how the over-reliance on animal models, methodological inconsistencies, and insufficient exploration of host and environmental factors hinders a unified understanding of the interconnected gut-brain pathways in AD.

### 3.1 Study limitations

#### 3.1.1 Over-reliance on animal models and limited human evidence

The field is dominated by preclinical studies utilizing transgenic mouse models (APP/PS1, SAMP8, ICV-STZ). While invaluable for mechanistic insight, these models poorly replicate the complexity of human gut-brain signaling, immune responses, and the multifactorial nature of sporadic AD. Species-specific differences in gut microbiota composition, lifespan, and neuroimmune pathways limit the direct translatability of findings (Lukiw et al., 2021; Su et al., 2023; Heydari et al., 2025). Human trials remain scarce and are often limited to small-scale, short-term pilot studies, as illustrated by one dietary intervention that was later retracted (Simopoulos et al., 2022). This heavy reliance on animal data creates a significant evidence gap between mechanistic promise and clinical efficacy.

#### 3.1.2 Short intervention durations and methodological heterogeneity

Most intervention studies, particularly in animals, are short-term, typically lasting 3–8 weeks. This fails to mirror the chronic, decades-long progression of AD and does not assess the long-term sustainability of interventions. For instance, while quercetin improved cognition in mice after 21 days, real-world preventive strategies would require years of sustained intervention (Westfall et al., 2019; Altendorfer et al., 2025). Furthermore, methodological heterogeneity, such as the use of varying probiotic strains, doses, and administration routes, complicates cross-study comparisons and meta-analyses. The strain-specific and route-dependent efficacy of interventions, exemplified by *Lactobacillus rhamnosus* in AD rats and the oral-specific effects of walnut peptide PW5, highlights the challenge of standardizing protocols (Li et al., 2022; Simopoulos et al., 2022).

#### 3.1.3 Inadequate consideration of confounding host and environmental factors

A major limitation is the frequent oversight of critical variables that shape the gut-brain axis in humans. Many preclinical studies use male-biased cohorts, overlooking documented sex-specific responses; for example, the probiotic VSL#3 reduced Aβ plaques only in female APP/PS1 mice, yet the underlying mechanisms for this disparity remain uninvestigated (Gao et al., 2019; Ruotolo et al., 2020; Pluta et al., 2022). Moreover, common comorbidities in AD patients, such as obesity, T2DM, and cardiovascular disease, are known to exacerbate gut dysbiosis *and* systemic inflammation, yet they are often controlled for inadequately or not at all in experimental designs. The profound influence of diet and medications (antibiotics, metformin) on gut microbiota is rarely accounted for, making it difficult to isolate the effect of the intervention itself and to establish clear causal relationships.

#### 3.1.4 Incomplete mechanistic insight and oversimplification of pathways

Many studies report correlations, such as between a rise in *Akkermansia* and reduced Aβ, without establishing definitive causality or elucidating the precise signaling pathways. For example, although *Akkermansia muciniphila* improves cognition, the neuroprotective role of its extracellular vesicles (EVs) and their specific cargo (miRNAs, proteins) are not fully understood (Ou et al., 2020; Cecarini et al., 2021; He et al., 2022; Ma et al., 2025). The mechanisms by which microbial metabolites like SCFAs cross the BBB and interact with neural receptors (FFAR2/3) remain poorly defined. There is also a tendency to investigate pathways in isolation, neglecting the complex crosstalk between, for instance, SCFA receptor signaling, TLR4 activation, and AhR pathways. Finally, the research focus has been disproportionately centered on amyloid and tau pathology, often neglecting other critical AD-related mechanisms such as neurovascular dysfunction, oxidative stress, and synaptic integrity (Linjuan et al., 2024).

### 3.2 Research gaps

#### 3.2.1 Need for robust human trials and mechanistic depth in human context

There is an urgent, unmet need for large-scale, long-duration randomized controlled trials (RCTs) in humans. Current evidence relies heavily on animal studies, with only a few early-phase trials testing human-derived probiotic cocktails (Kaur et al., 2020; Su et al., 2023). Future human trials must incorporate multi-omics profiling (metagenomics, metabolomics, proteomics) to move beyond correlation and validate biomarkers and signaling pathways within the human context. Key mechanistic questions to be addressed in human-relevant models include: How do microbial metabolites like TMAO and SCFAs cross the BBB? How does bacterial LPS directly interact with Aβ to drive aggregation and neuroinflammation? How do bacterial EVs transmit signals via the vagus nerve? (Kaur et al., 2020; Cecarini et al., 2021; Wang et al., 2021; Zhang K. et al., 2022; Chen Y. Y. et al., 2023).

#### 3.2.2 Development of personalized and stratified medicine approaches

The “one-size-fits-all” approach is unlikely to succeed given the high inter-individual variability of the gut microbiome. A critical gap is the development of personalized treatment strategies that account for host-microbiome interactions. This includes stratifying patients by genetic factors such as vitamin D receptor (VDR) polymorphisms and APOE4 status, as these can dramatically alter responses to interventions like vitamin D or omega-3 supplementation (Lawrence and Hyde, 2017; Ma et al., 2018; Rosa et al., 2020; Yang S. et al., 2020; Kaur et al., 2021; He et al., 2022; Wang et al., 2022; Prajapati et al., 2025). Genotype-stratified trials enrolling patients at the mild cognitive impairment (MCI) stage are needed to identify responsive subgroups and optimize therapeutic efficacy.

#### 3.2.3 Expansion of therapeutic targets and modalities

Therapeutic approaches must extend beyond amyloid-centricity to include tau pathology, neurovascular health, and sustained control of neuroinflammation. Research into synbiotic formulations (combinations of inulin and eicosapentaenoic acid [EPA]) represents a promising multi-target strategy that requires further exploration. Furthermore, long-term safety evaluations are essential, particularly regarding the risks of antibiotic-induced dysbiosis, the persistence of probiotic strains, and the potential for certain microbes like *Clostridium scindens* to produce neurotoxic secondary bile acids.

#### 3.2.4 Integration of multimodal interventions and early prevention strategies

A significant gap lies in understanding the synergy between gut-targeted therapies and other non-pharmacological modalities. Research is needed to study the combined effects of, for example, butyrate-producing probiotics and aerobic exercise, or between ketogenic diets and SCFA signaling (Alexandrov et al., 2020; Ruotolo et al., 2020; Wang et al., 2022; Altendorfer et al., 2025). Finally, the field must shift towards early prevention, investigating whether interventions in pre-symptomatic at-risk populations (APOE4 carriers) or even during critical developmental windows (perinatal microbiome programming) can engineer resilience against future AD pathology.

While non-pharmacological interventions targeting the gut microbiome hold significant potential, clinical translation is currently hindered by a nexus of limitations including poor model translatability, short study durations, methodological inconsistencies, and incomplete mechanistic insight. Future studies must prioritize well-designed human RCTs that incorporate deep phenotyping, focus on personalized protocols, and develop multimodal strategies that synergistically target the gut-brain axis at multiple levels to effectively modify the course of AD.

## 4 Prospective studies targeting the gut-brain axis in AD

Building on the compelling preclinical evidence and the critical gaps identified in Section 3, this section outlines a strategic roadmap for future research to translate gut-brain axis modulation into viable clinical strategies for AD. The priorities are designed to move beyond correlation to causation, bridge the translational gap between animal and human studies, and embrace the complexity of host-environment-microbiome interactions through personalized and multimodal approaches.

### 4.1 Human trials and dietary interventions

To address the critical lack of robust human evidence, large-scale, long-term RCTs are urgently needed. These trials should move beyond short-term pilot studies and investigate interventions with strong mechanistic backing, such as *Akkermansia muciniphila* supplementation, human-origin probiotic cocktails, or synbiotics, over periods of 12 to 24 months in cohorts with early AD or MCI. Outcomes must be comprehensive, including cognitive decline (ADAS-Cog), core AD pathology (via Aβ and tau PET imaging), systemic and central inflammatory cytokines, and gut permeability markers (zonulin). Such rigorous designs are essential for validating preclinical promises.

Concurrently, dietary interventions must be tested in high-risk populations, such as APOE4 carriers, with consideration for common comorbidities like obesity and T2DM. Prospective studies should evaluate combined regimens, for example, a Mediterranean-ketogenic diet hybrid supplemented with omega-3 EPA, for 18 months or longer. Primary outcomes should target the interplay between metabolism and brain health, including brain glucose metabolism (via FDG-PET), serum levels of gut-derived metabolites like trimethylamine N-oxide (TMAO), and longitudinal changes in gut microbiome diversity. This approach directly addresses the gap in long-term, human-relevant dietary studies that account for host genetics and comorbidity interactions.

### 4.2 Elucidating mechanistic pathways and developing personalized approaches

Future research must dissect the precise mechanisms of gut-brain communication to identify novel therapeutic targets. A key priority is to isolate and characterize bacterial EVs from beneficial strains like *Lactobacillus johnsonii* or *Akkermansia muciniphila*. Studies should track their biodistribution to the CNS in primate models using fluorescent labeling and analyze their cargo (miRNAs, proteins) to determine how they suppress microglial activation via NF-κB/TLR4 pathways or promote neuronal integrity.

Furthermore, the signaling of microbial metabolites must be delineated with greater precision. Employing FFAR2/3 knockout mice would allow researchers to dissect the specific contribution of SCFA receptor signaling to microglial function, particularly in Aβ phagocytosis via downstream pathways like NRF2/SOD1. This preclinical work should be integrated with clinical translation: collecting fecal samples from AD patients before and after SCFA supplementation for metatranscriptomic analysis can identify receptor-specific microbial gene expression changes, bridging mechanistic insight from bench to bedside.

Given the variability in treatment response, personalized approaches are paramount. Prospective trials should recruit patients with amnestic MCI (aMCI) and stratify them by genetic factors such as vitamin D receptor (VDR) SNPs (rs1544410) or APOE4 status. This allows for testing genotype-informed interventions, such as combinations of quercetin and vitamin D or specific probiotic formulations. These studies should monitor intermediary outcomes like serum 25(OH)D levels, gut microbiota shifts (Firmicutes/Bacteroidetes ratio), and hippocampal BDNF levels to understand how host genetics modifies therapeutic efficacy.

To operationalize precision medicine, future research must leverage artificial intelligence (AI) and machine learning. Developing algorithms that integrate baseline multi-omics data (metagenomics, metabolomics) can predict an individual’s response to interventions like FMT from young donors or specific flavonoid metabolites. The goal is to move from a universal protocol to a predictive model that guides therapy based on an individual’s unique microbiome and metabolic signature.

### 4.3 Integrated multimodal strategies and early intervention paradigms

The complexity of AD necessitates combinatorial approaches. Future studies should investigate synergistic interventions that target the gut-brain axis through multiple, reinforcing mechanisms. For instance, an “exercise-microbiome synergy” study could combine structured aerobic exercise with *Clostridium butyricum* supplementation in both APP/PS1 mice and AD patients, measuring synergistic effects on synaptic plasticity (long-term potentiation), colonic butyrate levels, and BBB integrity (measured by S100β). The hypothesis is that exercise amplifies the probiotic-induced increase in neuroprotective SCFAs.

Another innovative approach is a “diet-probiotic-device triad,” testing a combination of a ketogenic diet, the multi-strain probiotic VSL#3, and transcranial light therapy in mild AD. This would evaluate outcomes related to mitochondrial biogenesis (PGC-1α), NLRP3 inflammasome suppression, and fecal Akkermansia abundance, based on the rationale that diet-microbe interactions can be enhanced by direct neuromodulation.

Prospective research must also prioritize early intervention. This includes enrolling pre-symptomatic APOE4 carriers (cognitively normal adults aged 50–60) for long-term (5-year) interventions using agents like inulin prebiotic or curcumin, aiming to dampen neuroinflammation decades before clinical symptom onset. Primary outcomes should include plasma neurofilament light (NfL), hippocampal volume loss, and levels of immunogenic molecules like *Bacteroides fragilis* LPS.

Finally, the integration of digital health technologies is crucial for advancing monitoring and personalization. Prospective research should employ digital phenotyping platforms that combine wearable sensors (for sleep and activity), smartphone-based cognitive tests, and frequent fecal metabolomics (GC–MS). Using machine learning to analyze this dense longitudinal data can help predict individual disease trajectories and optimize interventions in real-time, creating a dynamic, closed-loop system for managing AD risk.

### 4.4 Concluding research outlook

Prospective research must bridge preclinical promise with clinical rigor by prioritizing large-scale human trials with multi-omics depth, elucidating the specific roles of bacterial EVs and metabolite-receptor interactions, personalizing interventions via host genetics and AI-driven microbiome phenotyping, and testing synergistic multimodal regimens. By targeting early disease stages and leveraging digital tools for monitoring, future studies can transcend Aβ-centric models and fully leverage the modifiable gut-brain axis as a pivotal target for the prevention and treatment of AD.

## 5 Conclusion

The evidence synthesized in this review firmly establishes the gut-brain axis as a central and modifiable pathway in AD pathogenesis. Moving beyond correlation, studies demonstrating that fecal microbiota transplantation from AD patients can induce cognitive deficits and neuropathology in healthy rodents provide compelling evidence of a causal relationship. The underlying mechanisms are multifaceted, centering on dysbiosis-induced compromise of the intestinal and BBB. This breach facilitates systemic inflammation and the influx of neurotoxic agents like LPS, while simultaneously disrupting the production of key microbial metabolites. The resultant deficit in neuroprotective SCFAs and indoles, coupled with an upsurge in pro-inflammatory mediators, directly fuels the core pathological triad of AD: chronic neuroinflammation, Aβ accumulation, and tau pathology.

Therapeutic targeting of this axis holds significant promise. Interventions such as specific probiotics (*Clostridium butyricum*), anti-inflammatory diets, polyphenols, and physical exercise have demonstrated efficacy in preclinical and early clinical settings by restoring eubiosis, enhancing barrier integrity, and suppressing neuroinflammatory cascades. However, the translation of these findings into mainstream clinical practice faces substantial hurdles. The field is currently constrained by an overreliance on animal models with limited translatability, a preponderance of short-term intervention studies, and a critical lack of large-scale, standardized human trials. Furthermore, methodological heterogeneity and an often-oversimplified view of microbiome-host interactions obscure precise mechanistic insights and hinder the reproducibility of outcomes.

To fully realize the therapeutic potential of the gut-brain axis, future research must be guided by a new paradigm. This entails a decisive shift towards large-scale, long-duration randomized controlled trials in human populations, with a particular focus on early disease stages. Achieving greater mechanistic depth through integrated multi-omics approaches is essential to delineate the precise signaling pathways, such as FFAR2/3, TLR4, and AhR, by which gut-derived metabolites influence brain health. Ultimately, the future of AD intervention lies in personalized medicine; strategies must account for host genetics, baseline microbiome composition, and comorbidities to develop effective, synergistic multimodal regimens. By addressing these critical gaps, modulation of the gut-brain axis can evolve from a compelling scientific concept into a tangible and powerful strategy for the prevention and treatment of AD.

## Glossary

AD: Alzheimer’s disease
ADAS-Cog: Alzheimer’s disease assessment scale-cognitive subscale
AhR: aryl hydrocarbon receptor
aMCI: amnestic mild cognitive impairment
Aβ: amyloid-beta
BBB: blood–brain barrier
BDNF: brain-derived neurotrophic factor
C3G: cyanidin-3-O-glucoside
CNS: central nervous system
DR3: death receptor 3
EPA: eicosapentaenoic acid
EVs: extracellular vesicles
FDG-PET: fluorodeoxyglucose positron emission tomography
FFAR2/3: free fatty acid receptor 2/3
FMT: fecal microbiota transplantation
GC–MS: gas chromatography–mass spectrometry
GLP-1: glucagon-like peptide-1
GPCRs: G-protein coupled receptors
GSK-3β: glycogen synthase kinase 3 beta
HDACs: histone deacetylases
IAA: indole-3-acetic acid
ICV-STZ: intracerebroventricular-streptozotocin
IKK: IκB kinase
IL-10: interleukin-10
KYNA: kynurenic acid
LPS: lipopolysaccharide
MCT: medium-chain triglycerides
MCI: mild cognitive impairment
MGnD: microglial neurodegenerative phenotype
miRNAs: microRNAs
NfL: neurofilament light chain
NF-κB: nuclear factor kappa-light-chain-enhancer of activated B cells
NLRP3: NACHT, LRR and PYD domains-containing protein 3
NRF2: nuclear factor erythroid 2–related factor 2
PET: positron emission tomography
PGC-1*α*: peroxisome proliferator-activated receptor gamma coactivator 1-alpha
PVLs: phenyl-*γ*-valerolactones
RCTs: randomized controlled trials
ROS: reactive oxygen species
SCFAs: short-chain fatty acids
SIRT1: sirtuin 1
SNPs: single nucleotide polymorphisms
SOD1: superoxide dismutase 1
SYN1: synapsin I
T2DM: type 2 diabetes mellitus
TLR4: toll-like receptor 4
TMAO: trimethylamine N-oxide
TNF-α: tumor necrosis factor alpha
VDR: vitamin D receptor
